# Analysis of Degradation Products of Biodegradable ZnMgY Alloy

**DOI:** 10.3390/ma16083092

**Published:** 2023-04-14

**Authors:** Cătălin Panaghie, Georgeta Zegan, Alina Sodor, Nicanor Cimpoeșu, Nicoleta-Monica Lohan, Bogdan Istrate, Ana-Maria Roman, Nicoleta Ioanid

**Affiliations:** 1 Faculty of Materials Science and Engineering, “Gh. Asachi” Technical University from Iasi, 700050 Iasi, Romania; 2 Faculty of Dental Medicine, “Grigore T. Popa” University of Medicine and Pharmacy, 700115 Iasi, Romania; 3 Faculty of Mechanics, “Gh. Asachi” Technical University from Iasi, 700050 Iasi, Romania

**Keywords:** ZnMgY alloy, biodegradable material, corrosion products

## Abstract

Biodegradable metallic materials are increasingly gaining ground in medical applications. Zn-based alloys show a degradation rate between those recorded for Mg-based materials with the fastest degradation rate and Fe-based materials with the slowest degradation rate. From the perspective of medical complications, it is essential to understand the size and nature of the degradation products developed from biodegradable materials, as well as the stage at which these residues are eliminated from the body. This paper presents investigations conducted on the corrosion/degradation products of an experimental material (ZnMgY alloy in cast and homogenized state) after immersion tests in three physiological solutions (Dulbecco’s, Ringer’s and simulated body fluid (SBF)). Scanning electron microscopy (SEM) was used to highlight the macroscopic and microscopic aspects of corrosion products and their effects on the surface. An X-ray energy dispersive detector (EDS), X-ray diffraction (XRD) and Fourier transform infrared spectroscopy (FTIR) provided general information about the compounds based on their non-metallic character. The pH of the electrolyte solution was recorded for 72 h during immersion. The pH variation of the solution confirmed the main reactions proposed for the corrosion of ZnMg. The agglomerations of corrosion products were on the micrometer scale, mainly oxides, hydroxides and carbonates or phosphates. The corrosion effects on the surface were homogeneously spread, with a tendency to connect and form cracks or larger corrosion zones, transforming the pitting corrosion pattern into a generalized one. It was noticed that the alloy’s microstructure strongly influences the corrosion characteristics.

## 1. Introduction

Biodegradable metals have emerged as promising materials for developing devices with possible medical applications. Their main purpose is to avoid potentially unwanted effects caused by permanent implants, and to eliminate the necessity of subsequent surgical interventions. A concrete definition for biodegradable materials would be “metals that are expected to corrode gradually in vivo, with an appropriate host response followed by the removal of corrosion products, then complete dissolution after the task for which it was designed has been completed” [1].

The three main classes of biodegradable materials are magnesium, zinc, and iron-based alloys. Research attention is focused on the gradual degradation and corrosion mechanism. The biomaterials that are created for implants are used to support tissues during healing, and thus can be designated for various medical applications. Compared to polymeric materials, biodegradable metals have a higher mechanical strength and better performance when used as cardiovascular stents and bone implants [2,3,4]. Numerous studies have presented the state-of-the-art technologies in the development of degradable biomedical implants based on biodegradable materials [5,6,7,8].

It has been observed that Mg ions released from biodegradable Mg-based implants have a positive impact on bone regeneration [9]. This type of alloy has attracted interest in the research field of in vitro and in vivo testing due to their good mechanical properties and adequate biocompatibility and biodegradability. A lot of research has been carried out on different types of Mg-based implants, such as pins, plates, or bone screws [10,11].

Unfortunately, the use of these kinds of metallic systems in vivo has led to major problems, limiting their capabilities to act as acceptable structural materials for biodegradable implants in practical applications. These obstacles are, in the case of Mg, the accelerated corrosion rate and the release of hydrogen that can lead to the premature loss of the mechanical integrity of the implant [12,13]. Biodegradable Fe-based alloys are also of great interest for medical applications due to their good biodegradability and biocompatibility. This type of alloy has excellent mechanical properties [14,15,16,17,18]. In the case of Fe-based alloys, some limitations have been observed, such as the degradation rate that is too slow for some clinical applications [19,20]. In research, Kraus et al. [20] observed that the iron-based alloy stent was almost intact after 360 days of implantation in a blood vessel. Because of the very slow degradation rate, physiological reactions between the tissue and implant can occur. Some reactions could be protein adsorption or tissue rejection. [21,22,23]. In the case of iron, it corrodes at an acceptable rate, but accumulates a bulky corrosion product that repels neighboring cells and organisms [24]. By adding noble elements, the degradation rate can be increased [25].

Zinc-based implants possess relatively low mechanical properties in vivo and low corrosion rates that can be problematic, and this limits their use as biodegradable implants [24,26]. Therefore, efforts are being made and research is being carried out to overcome these obstacles.

In terms of possible medical applications, the degradation of Zn and its alloys are mainly evaluated through in vitro and in vivo studies. The corrosion behavior of Zn-based materials is commonly evaluated by electrochemical and mass loss tests, determined in vitro. Standard potentiodynamic polarization (PP) and electrochemical impedance spectroscopy (EIS) analyses are commonly used in electrochemical corrosion testing. In previous years, the in vitro degradation of Zn-based materials has been investigated quite extensively through analyses using different corrosion media, such as Dulbecco’s phosphate buffered saline w/o calcium or magnesium (DPBS) solution, SBF, and Ringer’s solution [27,28,29,30,31]. To improve the suitability of Zn-based BMs for medical implants with respect to their mechanical performance, various approaches, including alloying, different forming techniques, and surface modification have been investigated [2,29,32]. Amongst these, Zn alloyed with Mg has been identified as a potential material for BMs.

Following the immersion of samples in different solutions during in vitro testing, and through the analysis of the surface of the material, corrosion zones can be observed with the formation of some reaction compounds, mainly oxides, formed during the corrosion process that will later lead to the zinc-based alloy degradation [33].

Alloying with chemical elements, such as Mg and Y, the Zn-based alloy forms compounds that delay the degradation process [34]. The use of ZnMg-based alloys, as well as alloying with Y, leads to the formation of intermetallic compounds, that contribute to increasing the corrosion resistance and mechanical properties, such as wear and tensile strength within acceptable limits, while preserving the biodegradability property for a longer time [35].

Through their study, Xiao et al. [28] showed that alloying Zn with up to 10% Mg content decreases the corrosion rate, compared to pure Zn, which allows the ZnMg alloy to heal the diseased tissue by preserving the mechanical integrity as long as necessary. It was found that an electrochemically inert layer of Mg_2_(OH)_2_CO_3_ was formed on the surface of ZnMg alloys to which the intermetallic phases of Mg2Zn11 and MgZn2 contributed [29]. It has been shown in studies that alloying Zn with 1% Mg leads to excellent strength and ductility properties (UTS ~250 MPa and ε ~12%) [4]. Other studies reported that Zn-3wt%Mg cast alloys have a very refined nanostructure and are corrosion resistant [4,36].

In this research, the corrosion behavior of a possible biodegradable alloy ZnMgY in different solutions (Dulbecco’s, Ringer’s and SBF) was analyzed. The analysis of the corroded surface was performed, the pH variation of the solution was evaluated, the corrosion rate based on mass loss was determined, and the corrosion compounds were identified.

## 2. Materials and Methods

The proposed experimental alloys were obtained from pure zinc (99.995 %wt) as well as ZnMg and ZnY main alloys with pure Y for correction by classical melting in an induction furnace (Inductro furnace with controlled Ar atmosphere) and graphite crucible [37]. All cylindrical ingots were subjected to heat treatment for chemical composition homogenization at 300 °C and controlled atmosphere (Ar) for 300 min. The alloying elements Mg and Y have been proposed for improving the mechanical properties of pure Zn, and preliminary studies have already been published [38].

The immersion tests were performed at 37 °C in three solutions, respectively, SBF (obtained in the laboratory), Dulbecco’s solution (without Ca and Mg), and Ringer’s solution (the last two were acquired from specialized laboratories, their chemical compositions are given in Table 1). The pH variation of the solution was registered per minute for 72 h, during the ZnMgY alloy immersion using Hanna HI98191 pH-meter equipment.

Samples with masses below 50 mg were prepared and cleaned with technical alcohol for a differential scanning calorimetric (DSC) analysis. The equipment used for the experiment was NETZSCH 200 F3 Maia. The apparatus was calibrated with Hg, Bi, In, Sn, and Zn standards. The temperature program consisted of heating, starting at room temperature (RT) and increasing to 450 °C with a 20 K/min heating rate, followed by cooling to RT with a 20 K/min cooling rate. A protective atmosphere of Ar and liquid nitrogen for the cooling process were used. DSC thermograms were evaluated with Proteus software version 4.8.5 using the tangent method to determine critical melting and crystallization temperatures and the linear baseline to determine the specific heat dissipation/absorption.

SEM (VegaTescan LMH II, SE detector, 30 kV power supply, 15.5 mm working distance) was used to investigate the surface of the alloy after the immersion tests. To identify the chemical elements (automatic and mapping modes) from the surface and their compounds that passed into the solution, EDS and a detector from Bruker (XFlash 6–10) with Esprit 2.2 software were used.

Following the immersion of the samples in SBF, the surfaces were inspected through XRD (Expert PRO-MPD system -XRD, Panalytical, Almelo, copper-X-ray tube (Kα-1.54°)) to identify the oxides and other compounds formed on the ZnMgY alloy at 25 °C (room temperature). The compounds extracted from the electrolyte solution were investigated using Fourier-transform infrared spectroscopy (FTIR). The infrared measurements were conducted on a Bruker-Vertex70 spectrometer (Bruker Optics, Ettlingen, Germany). Infrared absorption spectra (FTIR) were recorded on a Fourier transform spectrometer in the spectral range of 400–4000 cm^−1^ with a 4 cm^−1^ resolution.

The experimental activities conducted were based on the principles of health and safety at work, the use of compliant instrumentation and equipment, and adequate personal protective equipment [39].

## 3. Results

The objective of the proposed alloy was to improve the mechanical properties of pure Zn using alloying elements, such as Mg and Y. The experimental alloy proposed for analysis in this paper was obtained by studying the ZnMg, ZnY, and ZnMgY phase diagrams [40]. The structural and chemical aspects have been analyzed in a previous paper [37].

Research in the literature shows results confirming the substantial contribution of Mg content in zinc (1–3 wt%) that increases the volume fractions of the eutectic phase, improving the strength and hardness of pure Zn with a decrease in ductility [26,29]. The eutectic concentration for Mg in Zn solution is about 3 wt%; meanwhile, the maximum solubility of Mg in Zn is 0.1% [40]. Based on the biphasic diagram [40], the intermetallic compound formed in the ZnMg hypoeutectic zone (Mg ≤ 3 wt%) is Mg2Zn11, a hard and brittle eutectic phase that is distributed in the softer α-Zn matrix.

The ternary MgZnY alloy was analyzed by Gröbner et al. [40] with more than 10 ternary alloys in this system by different experimental techniques (DSC, SEM/EDXS, and TEM). Using their own experimental data and evaluation of the stoichiometry of the ternary phases reported in the literature [41,42,43,44,45,46,47,48,49,50,51,52,53,54,55,56,57,58,59,60], Gröbner et al. [40] calculated the liquidus projections and isothermal cross sections for the ZnMgY system [61].

In the literature, many of the reported ternary phases have been considered as metastable phases according to the work of Zhu and Pelton [60]. The slow transformation kinetics of ternary phases have been described in the literature with different notations and chemical compositions. In many MgZnY systems, a particular ternary phase corresponding to Mg12ZnY2 has been reported [56,60], and has been designated in the literature as phase X with the simplified composition Mg12YZn [41,47,60].

### 3.1. Differential Scanning Calorimetry (DSC) Analysis

**Figure 1:**
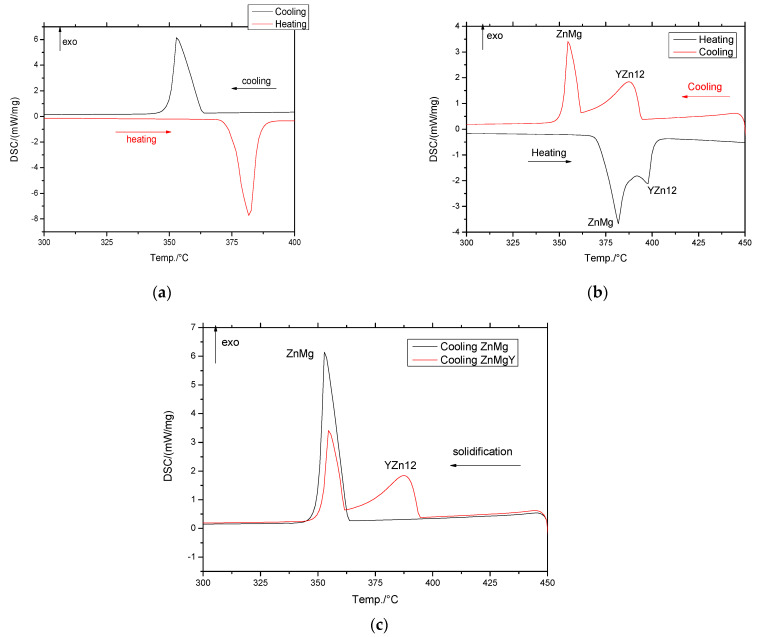
DSC curves of Zn3Mg and Zn3Mg0.4Y alloy: (**a**) melting and solidification of Zn3Mg alloy; (**b**) melting and solidification of Zn3MgY alloy; (**c**) solidification comparison of ZnMg with ZnMgY alloy.

The melting and solidification stages were investigated using DSC, and the heat flux variations by temperature are shown in Figure 1. Between ZnMg and ZnMgY alloys, a new peak can be observed in Figure 1 at melting and solidification, that can be attributed to the formation of YZn12 compound upon cooling, and melting upon heating. The intermetallic compound shows a different behavior than the solid solution with higher melting or solidification temperature (Figure 1b,c).

The main calorimetric parameters, extracted from the graphs (Figure 1), are shown in Table 2 and marked with M for melting and S for solidification. Even if the percentage of intermetallic compounds is low, their contribution to melting is important due to their high stability and properties. Even though the influence of the YZn12 phase on the corrosion resistance of Zn is unsubstantial, similar to other second phases, YZn12 particles play an extremely important role in the dynamic recrystallization—D.R.X. process of Zn alloys for the deformation processing stage. The increase and evolution of the deformation zone near the μm-sized YZn12 phase led to the concentration of stresses and promoted the D.R.X. process through the particle nucleation procedure [48]. For the smaller YZn12 phase (<1 μm), the effect is to inhibit the grain boundary displacement, an action that will negatively affect the deformation process. The additional elements decrease the melting temperature from 420 of pure Zn to 375 °C for the ZnMg alloy, and to 394 °C for the ZnMgY alloy, concluding that the addition of Y increases the melting temperature mainly through the formation of the YZn12 phase.

### 3.2. Immersion Tests

**Figure 2:**
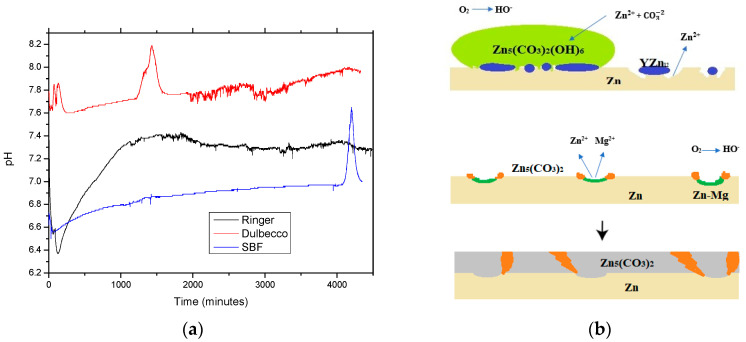
(**a**) pH variation of the immersion solution with ZnMgY and (**b**) a schematic of the corrosion process of the Zn-based alloy in an aqueous solution.

Typically, the corrosion of alloys is a complex process involving the classical corrosion process combined with electro-corrosion based on the different corrosion potentials of the elements, phases, grains, and their boundaries. In contact with an electrolyte solution, all materials, and especially alloys, undergo an exchange of ions and materials resulting in the oxidation of the metal surface and the subsequent corrosion concomitant with the pH variation of the solution. Chemical reactions occurring at the metal-liquid interface form acidic/basic radicals that pass into the solution and change the pH. Figure 2a shows the pH variations of different solutions during the immersion of ZnMgY alloy for 72 h.

Hydroxide-based compounds that are formed from the very beginning of the metal-solution interaction led to a constant increase in pH. Larger pH variations are due to a more pronounced exchange of ions and compounds, such as in the Dulbecco’s solution, between 1200 and 1500 min of immersion. Following the 2000 min period, for all three cases, a much more frequent exchange of ions between the immersed material and the solution is observed, with a high frequency of pH increases and decreases (constant formation and release of relatively small amounts of acids); but with a lower intensity of these variations, attributed to the stabilization of the corrosion process during this period under the protection of the corrosion compounds layer formed, and this has not yet transferred in massive amounts into the solution.

For all cases, an increase in the pH of the solution is observed, based on the formation of hydroxides. During the immersion period, decreases of the pH value are very often observed, due to localized corrosion, accompanied by the formation and release of acid in the solution. The main reactions of a Zn-based alloy in contact with an aqueous solution are presented in the following equations [61] and are shown schematically in Figure 2b.

2H_2_O + 2e^−^ → H_2_ + 2OH^−^ (1)

2H_2_O + O_2_ + 4e^−^ → 4OH^−^ (2)

Zn^2+^ + 2OH^−^ = Zn(OH)_2_ (3)

Zn → Zn^2+^ + 2e^−^ (4)

The common compounds formed in this case are oxides—ZnO, hydroxides—Zn(OH)_2_ and Zn_2_(OH)_8_Cl_2_·4H_2_O, phosphates—Ca_10_(PO_4_)_6_(OH)_2_, and Ca_3_(PO_4_)_2,_ and carbonates—Ca_10_(PO_4_)_6_(OH)_2_, Ca_3_(PO_4_)_2_, CaCO_3_, and Zn_5_(CO_3_)_2_(OH)_6_ [62,63,64,65,66,67]. The typical zincite compound is present over time on ZnMg and ZnMgY corroded surfaces [68]. Y is present in a small percentage in the alloy and its influence on the corrosion properties is almost insignificant [21,22]. During immersion, two compounds have been identified from the early stages, hydrozincite and skorpionite (Ca_3_Zn_2_(PO_4_)_2_CO_3_(OH)_2_·H_2_O) [69], which appear from the first hours of immersion, and are removed from the surface by the third day of immersion. Based on the results of Nazarov and Thierry [70], zinc carbonate and hydrozincite were the major corrosion compounds for the ZnMg alloy at the first atmospheric or aqueous contact. During immersion, two early-stage compounds, hydrozincite (Zn_5_(OH)_6_(CO_3_)_2_) and skorpionite (Ca_3_Zn_2_(PO_4_)_2_CO_3_(OH)_2_·H_2_O), were identified, which appear as early as the first hours of immersion, and are removed from the surface by the third day of immersion [71].

The evidence of simonkolleite and hydroxyapatite (HA) at the beginning of the immersion period can be confused with the formation of hydrozincite and skorpionite, respectively, being coincident.

**Figure 3:**
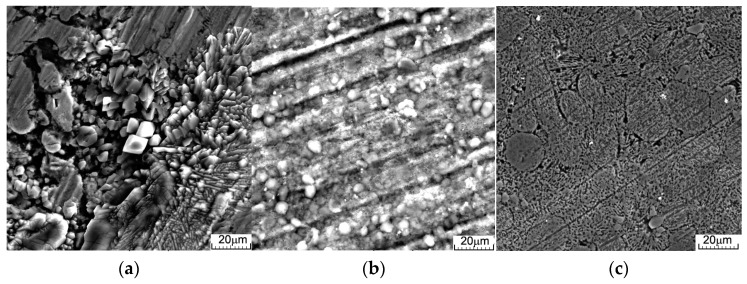

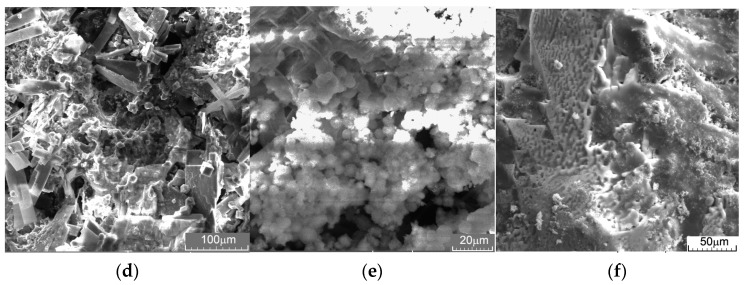
SEM images after immersion in Dulbecco’s, Ringer’s, and SBF of: (**a**–**c**) ZnMgY alloy surface; (**d**–**f**) the compounds resulted after immersion and air drying.

Surface images of the samples after immersion and ultrasonic cleaning, and the compounds extracted from each solution are shown in Figure 3. Sizing of the corrosion pits at the micro and nanoscale using VegaTc software version 3.5.0.0 gives a minimum value of 50 nm and a maximum value of 1.24 μm in diameter (average of ~600 nm ± 50 nm standard deviation), showing that the degraded parts are very small and cannot cause blockages of veins or major organs during their removal from the body. Simultaneously, these small holes (Figure 3c), can merge and cause larger pieces of material to detach. The type of corrosion and formation of compounds on the surface depend on the type of solution (Figure 3a–c) and degradation will occur governed by different mechanisms. In real cases, a combination of all of these types of degradation is involved in the corrosion and alloy degradation process.

**Figure 4:**
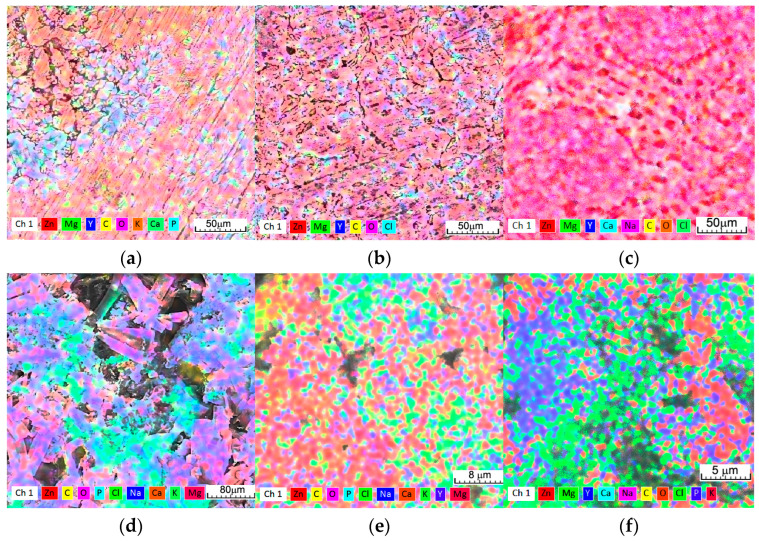
EDS mappings of the identified elements: (**a**–**c**) on the surface of ZnMgY alloy after immersion (Dulbecco’s, Ringer’s, and SBF); and (**d**–**f**) the distributions of the main elements on the compounds recovered from the solutions.

Following three days of immersion in Dulbecco’s solution, the simonkolleite can be observed on the surface (Figure 3a), and for the Ringer’s immersion, hydrozincite and hydroxyapatite are observed even after ultrasonic cleaning. In the case of SBF immersion, no corrosion products are observed—the sample is generally attacked with small pitting holes, and more aggressive corrosion can be observed at the grain boundary [72]. The identification of light spherical aggregates (Figure 3) on the alloys after 3 days, with phosphorus and calcium maps (Figure 4), could be related to the growth of HA. From the mapping images (Figure 4a–c), O-rich areas can be considered to be related to cathodic sites and Cl-rich areas with the anodic ones.

On the corrosion products of ZnMgY, the typical lamina-like structures could be detected, and the chloride map in Figure 4 confirmed that the simonkolleite compound was present on the corrosion layer from the start of the immersion. Simonkolleite growth on the surface is a less porous material that slows down the corrosive process of the layer formed.

Mass loss after the immersion in all three solutions was recorded after three days, and the corrosion rate was calculated [73]. The results are given in Table 3. On the surface, a mass gain was observed in all three cases, with a plus for Dulbecco’s solution, where there were more corrosion products and electrolyte compounds passing to the surface. The least reactive case is the sample in SBF (the solution with the most compounds in the formula), where better passivation probably occurred based on the oxidation process and the stability of the compounds formed at the surface. Simultaneously, after ultrasonic cleaning, a mass loss was observed in all cases, with a very similar corrosion rate in Ringer’s and SBF solutions. Comparing the corrosion rates, a tenfold lower rate was obtained in Dulbecco’s solution, where the phosphate-based layer formed on top of the material provides better protection at the beginning of the corrosion process. The differences in degradation rates may be due to the chemical composition of the electrolyte solutions. Ringer’s and SBF solutions possess an excess of Cl^-^ ions, that can transform the layer formed by Mg(OH)_2_ into more soluble MgCl_2_, increasing the corrosion rate. We can conclude that SBF affects the surface in depth more than Dulbecco’s solution.

The chemical composition of the corrosion products after immersion and ultrasonic cleaning found on the samples is shown in Table 4. For the basic compounds, zinc carbonate, hydrozincite, and oxides were the main corrosion products for ZnMg and ZnMgY alloys. Depending on the immersion solution, chloride or phosphate compounds were identified. Salts occur mainly after the immersion in Ringer’s and SBF solutions. No phosphate compounds were identified on the surface after immersion in Ringer’s solution, and only the chemical composition of the compounds was observed, probably after intense interaction between the solution and the compounds that passed from the surface. Simultaneously, no Y-element was identified in the compounds formed after immersion in Dulbecco’s solution, meaning that the Y-based phase has a higher strength in Dulbecco’s solution compared to the SBF solution.

A high carbon content (EDS data usually show a large error in C identification) overlapping with a typical grey crystal structure (Figure 4), detected three days after immersion, was consistent with increased hydrozincite on the surface of the ZnMgY alloy.

**Figure 5:**
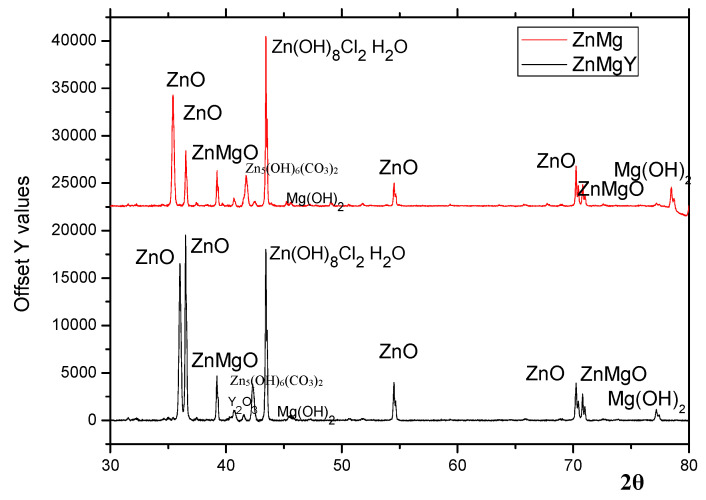
XRD patterns of ZnMg and ZnMgY alloys after 72 h of immersion in SBF.

The crystalline quality of the surfaces was analyzed using XRD determinations. Diffraction peaks were recorded at 2θ angle. The XRD pattern is shown in Figure 5 and was performed on the corroded surface of ZnMg and ZnMgY alloys after immersion in SBF for three days and ultrasonic cleaning in technical alcohol for 60 min. It was observed that the main corrosion products passed from the surface into the solution during the immersion and cleaning steps. As the surface was covered with ZnO, which was the main corrosion product, a protective layer was formed at the beginning [74]. The zincite layer was repeatedly formed, and was penetrated by various ions in the electrolyte solution. Following continuous growth of the corrosion layer and its removal from the surface, this oxide layer formed again, protecting the alloy for a short amount of time.

In addition to ZnO peaks, several brucite (Mg(OH)_2_) peaks were identified on both diffractograms [75]. A cluster of peaks attributed to zinc carbonate hydroxide (Zn_5_(CO_3_)_2_(OH)_6_) was also observed [76]. A small 2θ peak around 45.5 can be attributed to Y_2_O_3_, even though the percentage of Y is low in the material [77]. Different forms of zinc chlorides (Zn(OH)_8_Cl_2_·H_2_O) or Zn-carbonate formation were observed in the top layers, formed mainly from corrosion products, including the Zn3MgY sample, zincite (ZnO), or its hydrated forms. From the EDS and XRD analyses, it appears that Zn-based corrosion compounds are mainly oxides and mixed hydroxides, carbonates, and chlorides (Figure 4 and Figure 5). Mg and Zn have very different standard potentials, namely −2.372 and −0.762 V, respectively [78]. The higher corrosion potential of ZnMg alloys (−0.98 V versus −1.55 V for ZnMgY) leads to slower corrosion. The corrosion rate is also influenced by the high hydrogen overpotential of Zn, that suppresses hydrogen evolution on its surface. The cathodic current is limited by diffusion, which controls the reduction of dissolved oxygen by the cathodic reaction.

### 3.3. Phase Composition of the Corrosion Products

**Figure 6:**
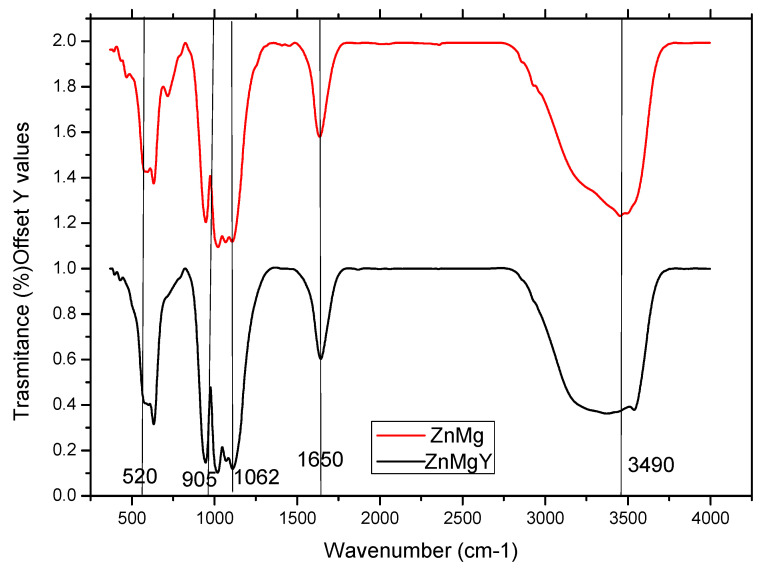
FTIR spectra of the corrosion compounds from ZnMg and ZnMgY samples after immersion in SBF solution. The vertical line explaine the exact values of the peaks.

The nature and type of corrosion products were investigated by FTIR spectroscopy. Infrared spectra of the corrosion compounds/products extracted from Dulbecco’s solution after the immersion of ZnMg and ZnMgY samples are shown in Figure 6. Higher peaks with maxima at 3500–3400 cm^−1^ are attributed to vibrations and rotations of H_2_O molecules and the OH^-^ group. Few minor differences between the spectra can be observed in the wavenumber range from 400 to 1750 cm^−1^. The results in Figure 6 show that a band with the peak centered at 520 cm^−1^ correlates with the zinc oxide (zincite) bond [79].

The corrosion product peaks in both alloys have a large peak at 1650–1250 cm^−1^ due to the bending vibration band of the CO^2-^_3_ compound. The peaks at 1300 and at 850 cm^−1^ are attributed to hydrozincite (Zn_5_(OH)_6_(CO_3_)_2_) [79,80]. The results confirmed the chemical determinations shown in Table 4 and the maps in Figure 4.

Peaks at 905 and 720 cm^−1^ indicate simonkolleite (Zn_5_(OH)_8_Cl_2_·H_2_O) in the corrosion products. Thus, some compounds in the corrosion products on zinc were hydrozincite and simonkolleite. Using the FTIR technique, no sodium carbonate was identified on ZnMg and ZnMgY alloys. However, through FTIR experiments, no Mg-based corrosion products were identified on the surface of ZnMg and ZnMgY alloys, based on a low weight percentage of Mg in the corrosion compounds (Table 4).

Simonkolleite is known to be a complex and stable material at lower pH values, usually characteristic for anodic sites, and hydrozincite is stable at higher pH values for cathodic sites [70]. The alternating formation of these compounds is confirmed by the pH variation of the solution, as shown in Figure 2a. Being in contact with a solution with salts (all three solutions tested have NaCl in their composition), the area near the NaCl deposits became a cathodic zone spreading toward the ZnMg passive solid solution. The distinct distribution of Cl and O (Figure 4b), as well as the lack of hydrozincite at the NaCl deposition sites, confirms the total separation of cathodic and anodic reactions on the surface of the ZnMgY alloy. Based on the fact that hydrozincite has a lower protective role compared to simonkolleite [81], it appears that the chemical composition of zinc corrosion phases is irrelevant for the corrosion performance of ZnMgY alloy. The participation of Mg and Y in the Zn-based solid solution and the compounds formed in the alloy show selective corrosion of the elements and the appearance of different corrosion compounds. The oxygen reduction is prevented, probably on the basis of a lower electron transfer rate related to the corresponding insulation properties. The main purpose of the corrosion layer is to limit the rate of the anodic reaction by modifying the corrosion potential of ZnMgY alloys near the reversible potential of Zn, and by attenuating the potential reduction along the alloy-element oxide boundary with the anodic dissolution overstrain.

## 4. Conclusions

The corrosion compounds formed in three different electrolytes by the ZnMgY alloy are based on the interactions between the alloy and the solution, ion exchange, and corrosion potential of the component phases. The following conclusions were drawn:

– The main chemical degradation compounds are zincite, simonkolleite, skorpionite, hydrozincite, and some small traces of brucite;
– Corrosion sites on the surfaces start from a few nanometers and can grow to micrometer sizes;
– The degradation rate is similar for Ringer’s and SBF solutions and is much lower (10 times) in Dulbecco’s solution;
– XRD and FTIR results of the corrosion compounds confirm the formation and detachment of zincite (ZnO) and complex compounds, such as hydrozincite or simonkolleite;
– For a proper characterization of the corrosion compounds, it is suggested to perform daily tests from the first hour of immersion up to fourteen days, separately, for each type of immersion solution.

## Figures and Tables

**Table 1:** Chemical composition of the immersion medium [g/L].

Chemical Composition [g/L]	NaCl	KCl	CaCl_2_	KH_2_PO_4_	Na_2_HPO_4_·7H_2_O	Na_2_SO_4_	NaHCO_3_	1.0 M HCL	MgCl_2_·6H_2_O	K2HPO_4_·3H_2_O	((HOCH_2_)_3_CNH_2_)
SBF	8.03	0.22	0.29	-	-	0.07	0.35	39	0.31	0.23	6.11
Dulbecco’s (DPBS)	8.05	0.20	-	0.2	2.16	-	-	-	-	-	-
Ringer’s	6.5	0.42	0.25	-	-	-	0.20	-	-	-	-

**Table 2:** DSC results of Zn, ZnMg, and ZnMgY (different Y percentages: V: 1.0; IV: 0.8; III: 0.6; II: 0.4; I: 0.2 wt%Y) alloys.

Sample		Melting				Solidification			YZn12 Compound			
	Sample Mass [mg]	M_s_ [°C]	M_50_ [°C]	M_f_ [°C]	DH_heating_ [J/g]	S_s_ [°C]	S_50_ [°C]	S_f_ [°C]	S_s_ [°C]	S_50_ [°C]	S_f_ [°C]	DH_cooling_ [J/g]
Zn	11.7	420.5	424.9	426.1	−317.3	420.0	415.6	414.7	-	-	-	-
Zn3Mg	33.0	375.5	382.1	385.6	−166.0	363.0	353.0	350.6	-	-	-	-
ZnMgY_V	50.0	370.9	387.1	403.8	−170.1	356.7	345.8	339.2	382.9	373.5	357.0	134.2
ZnMgY_IV	37.7	378.2	392.1	396.5	−168.9	343.5	346.6	359.4	361.7	363.0	372.6	128.8
ZnMgY_III	50.0	377.9	386.1	389.9	−151.2	361.0	349.5	347.1	376.1	369.1	362.0	120.4
ZnMgY_II	38.2	394	398	400	−157.1	361.4	354.8	352.1	394.0	387.5	374.3	142.1
ZnMgY_I	35.8	368.5	383.4	390.4	−152.9	360.2	350.4	347.6	381.1	374.5	362.7	129.4

**Table 3:** Mass variation and corrosion rate of ZnMgY alloy in three different solutions (3 days immersion).

Sample Zn3Mg0.4Y/Solution	Ringer’s	SBF	Dulbecco’s
Initial mass (mg)	6739.4	15,607.1	4580.6
After immersion (mg)	6743.6	15,609.2	4589.3
	(+4.2)	(+2.1)	(+8.7)
After ultrasound cleaning (mg)	6734.7	15,595.7	4580.3
	(−4.7)	(−11.4)	(−0.3)
Corrosion rate (mm/year)	0.10	0.11	0.01

Sample areas: in Ringer’s = 7.7 cm^2^, SBF = 17.1 cm^2^, and Dulbecco’s = 5.7 cm^2^.

**Table 4:** Chemical composition (EDS detector determination using element list mode) for the alloys’ surfaces after immersion in the tested solutions (Dulbecco’s, Ringer’s, and SBF) and for the compounds that passed into the solution.

Element/Sample	O%		C%		Na%		Cl%		Zn%		P%		K%		Mg%		Ca%		Y%	
	wt%	at%	wt%	at%	wt%	at%	wt%	at%	wt%	at%	wt%	at%	wt%	at%	wt%	at%	wt%	at%	wt%	at%
in Dulbecco’s	29.29	59.82	-	-	-	-	-	-	61.42	30.69	8.22	8.67	0.26	0.21	0.3	0.40	-	-	0.52	0.19
CpS (Dulbecco’s)	32.78	48.79	-	-	25.63	26.55	20.51	13.77	12.62	6.18	6.18	4.75	1.91	1.16	0.39	0.38	-	-	-	-
in Ringer’s	23.54	45.96	8.21	21.35	-	-	0.37	0.33	66.96	31.99	-	-	0.05	0.05	-	-	0.13	0.1	0.87	0.31
CpS (Ringer’s)	20.38	21.18	46.92	64.93	5.86	4.24	10.31	4.83	12.8	3.26	-	-	2.1	0.89	0.01	0.01	1.55	0.64	0.07	0.01
in SBF	12.84	27.43	6.15	17.5	12.24	18.2	0.02	0.02	66.97	35.02			1.07	1.5	0.11	0.13			0.56	0.22
CpS (SBF)	25.01	23.21	55.08	68.1	3.58	2.31	11.75	4.9	1.97	0.44	0.71	0.34	0.76	0.29	-	-	0.9	0.33	0.24	0.05
EDS err.%	1.5/5		3/4		0.35/1.5		1.5/1.5		0.15/0.2		0.05/1.2		0.07/0.2		0.04/0.1		0.05/0.1		0.1/0.2	

CpS: compounds passed into the solution after immersion; Det. error: sample/compounds; StDev. (10 determinations on a 1 mm^2^ area): O ± 2.1; C: ±1.1; Na: ±0.75; Cl: ±0.9; Zn: ±0.75; P: ±0.5; K: ±0.25; Mg: ±0.1; Ca: ±0.2; and Y: ±0.1.

## Data Availability

All data presented in this study are contained within the article.
